# Sex specific inflammatory profiles of cerebellar mitochondria are attenuated in Parkinson’s disease

**DOI:** 10.18632/aging.103937

**Published:** 2020-08-27

**Authors:** Thomas L. Ingram, Freya Shephard, Sarir Sarmad, Catherine A. Ortori, David A. Barrett, Lisa Chakrabarti

**Affiliations:** 1 School of Veterinary Medicine and Science, University of Nottingham, Sutton Bonington, UK; 2 Centre for Analytical Bioscience, Advanced Materials and Healthcare Technologies Division, School of Pharmacy, University of Nottingham, Nottingham, UK; 3 MRC Versus Arthritis Centre for Musculoskeletal Ageing Research, UK

**Keywords:** inflammation, mitochondria, cerebellum, Parkinson's disease

## Abstract

Response to inflammation is a key determinant in many diseases and their outcomes. Diseases that commonly affect older people are frequently associated with altered inflammatory processes. Neuroinflammation has been described in Parkinson's disease (PD) brain. PD is characterized by the loss of dopaminergic neurons in the substantia nigra pars compacta and at the sub-cellular level, mitochondrial dysfunction is a key feature. However, there is evidence that a different region of the brain, the cerebellum, is involved in the pathophysiology of PD. We report relative levels of 40 pro- and anti-inflammatory cytokines measured in PD and control cerebellar mitochondria. These data were obtained by screening cytokine antibody arrays. In parallel, we present concentrations of 29 oxylipins and 4 endocannabinoids measured in mitochondrial fractions isolated from post-mortem PD cerebellum with age and sex matched controls. Our oxylipin and endocannabinoid data were acquired via quantitation by LC-ESI—MS/MS. The separate sample sets both show there are clearly different inflammatory profiles between the sexes in control samples. Sex specific profiles were not maintained in cerebellar mitochondria isolated from PD brains.

## INTRODUCTION

Though Parkinson’s disease (PD) is a single diagnosis, it presents as a heterogeneous disorder with some patients experiencing a more akinetic phenotype with prevailing rigidity where others may be less affected by rigidity but have a more tremor dominant disease [1]. The measurement of these different forms is now possible using MRI and reveals that certain neural loops, for example a ‘cerebello-thalamo-cortical loop’ can be associated with specific symptomology [2, 3]. There is mounting evidence to justify extending the study of PD from the basal ganglia to include connecting pathways to the cerebellum [4, 5]. However, moving our attention to the cerebellum where there is no overt cell loss warrants a careful understanding of what is happening in this region of the brain at a molecular and cellular level; this fits with recent proposals that the cerebellum is due for greater attention in the field of PD [5–7].

Mitochondrial dysfunction is a key feature of ageing and neurodegenerative disease [8, 9]. The link between mitochondrial dysfunction and disease is particularly well described in PD where it has been linked to both familial and sporadic forms. Mitochondria in PD have been shown to undergo bioenergetic changes, DNA mutations, misregulation of fission and fusion and dynamics more generally; mutations in mitochondrial proteins Parkin and PINK1 are implicated in PD [10]. A direct link between parkin and PINK1 and the STING inflammatory pathway connects pathways central to mitochondrial dynamics and neuroinflammation [11, 12]. The alpha-synuclein protein is regarded as central to PD pathology and has been implicated in neuroimmune response while also being involved in mitochondrial dysfunction. Mitochondrial associated membranes (MAMs) and mitochondrial damage-associated molecular patterns (mtDAMPs) also directly connect mitochondria in processes leading to changes in inflammatory response [13, 14].

In PD it is accepted that more males than females have the disease, with some studies calculating a nearly 2:1 ratio between sexes [15]. It is certainly possible that oestrogen may offer neuroprotection in females but direct evidence for this in PD is not yet forthcoming [16]. However, not only are females less likely to be diagnosed with Parkinson’s disease but sex differences are apparent in aspects of symptomology associated with this disease, for example swallowing disturbances, and dementia are associated more with male patients. Furthermore, normal human basal ganglia are sexually dimorphic, which might influence the onset and progression of PD [15]. When treating PD, it is apparent that there are sex differences in bioavailability of levodopa (the most potent medication for PD) even in much older post-menopausal patients. Females retain a higher availability for the drug than males with matched disease duration [17]. Sex differences in disease susceptibility and phenotype are evident in the clinic, but the molecular explanations for these are not established.

Lipid profiling by mass spectrometry allows the absolute quantitation of biologically active molecules in complex tissue preparations [18]. Oxylipins are a group of metabolites produced via oxidation of polyunsaturated fatty acids (PUFAs) [19], they have been linked to pathways of inflammation, pain and ageing [20–22]. In PD mis-folding of the protein alpha-synuclein is associated with dopaminergic cell death in the *substantia nigra*. Studies of the interaction of alpha-synuclein with PUFAs has indicated a protective role for alpha-synuclein in the inhibition of harmful oxidation reactions which affect the PUFAs [23, 24].

Arachidonic acid (AA) signalling is upregulated in the caudate-putamen and frontal cortex of unilaterally 6-hydroxydopamine (6-OHDA) lesioned rats, a model for asymmetrical PD [25]. Arachidonic acid and its derivatives are modulated in inflammation and oxidative stress and these are factors associated with induction of neurodegeneration in age-related neurological disorders [26]. Signs of inflammation are found in many neurodegenerative diseases and can be attributed to interactions between glial and neuronal cells, activated by microglia and astrocytes [27, 28]. Pro-inflammatory cytokine contributions to the inflammasome associated with PD could be potential targets for therapy [29, 30]. However, the role of inflammation and the specific molecular signatures found in healthy ageing versus neurodegeneration must be delineated and understood throughout the brain, before therapies to regulate cytokine production can be promoted [31, 32].

Our study looked at molecular markers of inflammation measured in mitochondrial fractions derived from cerebellum to reveal whether there is a disease related process that connects with PD in this part of the human brain.

## RESULTS

### Variability in cytokine and oxylipin levels characterize the female control group

**Figure 1:**
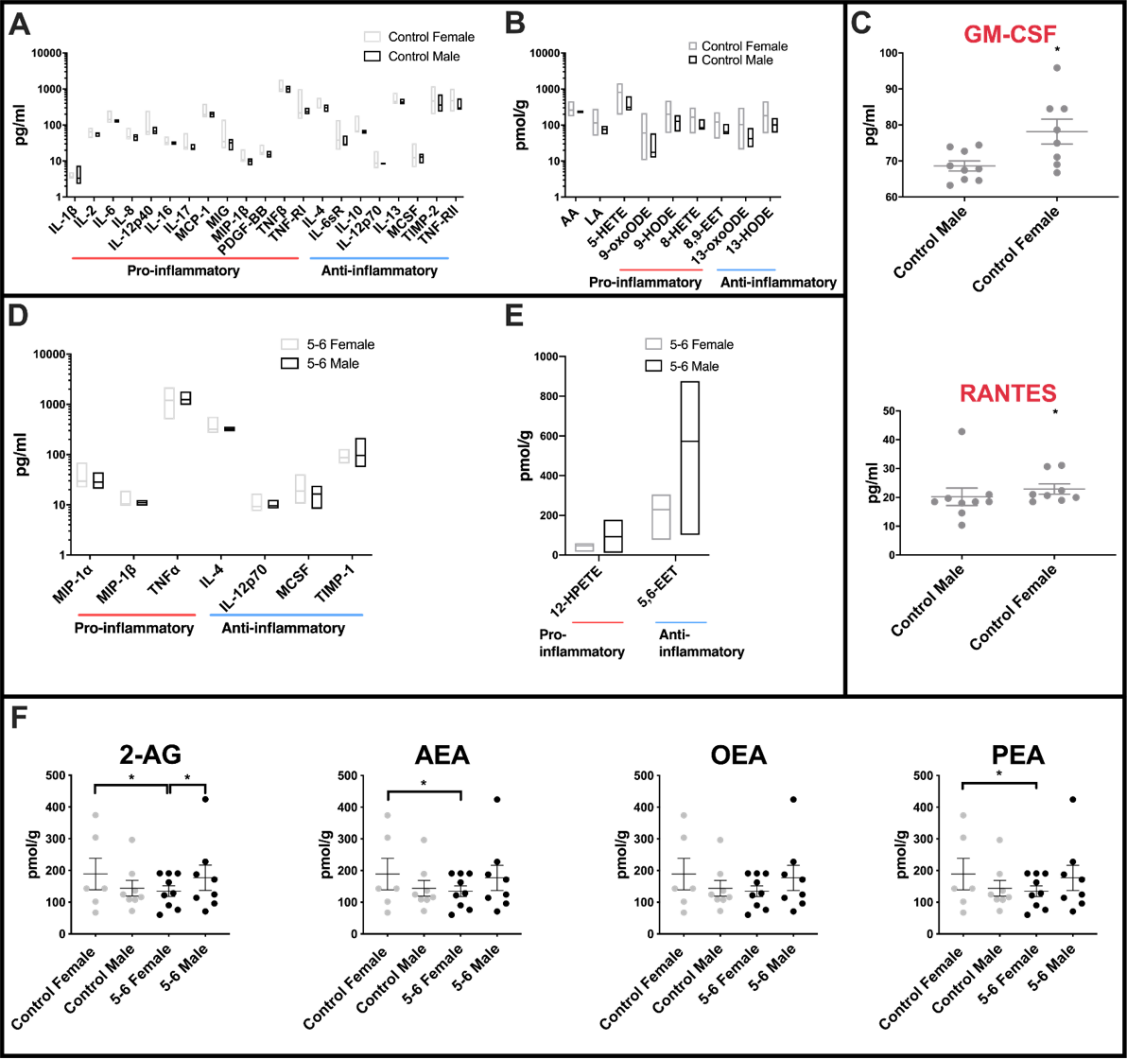
**Sex differences in inflammatory profiles of cerebellar mitochondria.** (**A**) Cerebellar mitochondria of female controls have highly variable inflammatory cytokine expression compared with male controls. Twenty-one inflammatory cytokines showed significantly different variance between female and male controls. Twenty of these were significantly more variant in females. Control male n=9; control female n=8. (**B**) Oxylipin variance is significantly greater in cerebellar mitochondria of female controls than male controls. Female controls have significantly greater variance in nine oxylipins. Male control cerebellar mitochondrial oxylipin levels are less variant within the group. Control male n=5; control female n=5. (**C**) Two inflammatory cytokines are significantly increased in female compared to male controls. RANTES and GM-CSF were significantly increased in mean levels in female controls compared to male controls. Control male n=9; control female n=8. (**D**) Cerebellar mitochondria from PD Braak 5-6 females have greater variability in inflammatory cytokine expression than the Braak 5-6 males. Six of seven significant inflammatory cytokines showed higher variance in the PD Braak 5-6 female group than PD Braak 5-6 males. PD Braak 5-6 male n=10; PD Braak 5-6 female n=9. (**E**) PD Braak 5-6 males and females show little variance in all but two cerebellar mitochondrial oxylipin levels. Two oxylipins have significantly higher variance values in PD Braak 5-6 males than females. PD Braak 5-6 male n=5; PD Braak 5-6 female n=5. All samples were age matched. A, B, D and E present inflammatory cytokine and oxylipins with significantly different variances (*f*-test). Refer to Supplementary Tables 4 and 6 for *f* values of oxylipins and inflammatory cytokines, respectively. Box plots display interleaved high and low. The horizontal line represents the mean. C shows significantly altered cytokines. Red title font represents pro-inflammatory cytokine. Displayed are mean levels ± SEM. (Mann-Whitney U-test). Refer to Supplementary Table 5 for *p* values. (**F**) Endocannabinoid variance is reduced in PD Braak 5-6 females. Female control group has heterogenous quantities of 2-AG, AEA and PEA compared with PD Braak 5-6 female group. Males do not show significant variation in endocannabinoid levels. Statistical analyses were carried out using GraphPad Prism (*f-*test). No significant differences were seen between the means of the groups (Kruskal-Wallis test with multiple comparison’s). Braak 5-6 male n=8; Braak 5-6 female n=9; control male n=8; control female n=6. All samples were age matched. Plots display mean ±SEM. Bars above plots represent statistically significant differences. Refer to Supplementary Table 7 for *p* and *f* values.

Highly variable inflammatory cytokine expression was measured across female control cerebellar mitochondria when compared with the equivalent male control group (Figure 1A). Of the forty cytokines interrogated on the slide arrays, twenty-one inflammatory cytokines had different variance (*f* -test) between female and male controls, only one of these (IL-1β) had a greater variance in males. Both pro- and anti-inflammatory molecules were largely found to show greatest variance in female control groups (for comprehensive graphs see Supplementary Figure 2 and datasets Table 6). Several very highly significant differences in pro-inflammatory molecules were found including IL-12p40, MIG and TNFRI (for abbreviations of molecules measured throughout see Supplementary Table 2). Very highly significant differences in variation were also found in anti-inflammatory cytokines including IL-10 and IL-12p70. Measurements of mean levels identified pro-inflammatory cytokines RANTES (*p=0.007*) and GM-CSF *(p=0.0418)* as significantly higher in female controls when compared with age-matched male control cerebellar mitochondria (Figure 1C).

Significant differences in variance between female and male control oxylipin concentrations were found in AA, LA and pro-inflammatory 5-HETE, 9-oxoODE, 9-HODE, 8-HETE, 8,9-EET, and also anti-inflammatory 13oxoODE and 13-HODE (Figure 1B – and for comprehensive graphs see Supplementary Figure 1 and datasets Table 4). Again, the female control group showed the greater levels of variance in each case.

### Fewer sex differences are found in cytokines and oxylipins of PD Braak 5-6 cerebellar mitochondrial fractions

Comparison of cerebellar mitochondria isolated from severely affected brain tissues shows fewer differences between the sexes. However, some molecules still present with different variation in levels. Cytokines measured with different variance between the sexes were pro-inflammatory MIP1α, MIP1β and TNFα which all showed a higher variance across the female Braak 5-6 group (Figure 1D and see Supplementary Table 6 for *f*-test data). Anti-inflammatory cytokines IL-4 and IL-12p70 showed a similar pattern of higher variance in female samples. For MCSF and TIMP-1 the male group has a greater degree of variation in cytokine levels than that found in females.

Of the oxylipins measured, just two show a difference in variation between the sexes in PD Braak 5-6 groups, both have a greater variation across the male group PD Braak 5-6 (Figure 1E). 12-HPETE an anti-inflammatory oxylipin, is found to have a higher variation across the male PD Braak 5-6 group, this is also the case for the only other oxylipin to show a difference, 5,6-EET (see Supplementary Table 4 for *f*-test data).

### Endocannabinoid levels show greater variance in the control female group

Though there are multiple reviews and studies on the effect of endo/cannabinoids on brains of mammalian models of PD [33, 34], the measurement of endocannabinoid species in human post-mortem PD brain has not been reported. Reflecting the datasets gathered from the oxylipin and cytokine measurements, we found that there was no mean difference between the four groups in the endocannabinoid species that were analysed (Figure 1F and Supplementary Table 7). However, once again the *f*-test showed there was a significant difference in variation when comparing the female group data with the PD Braak 5-6 female group in 3 of the four endocannabinoid species, namely 2-AG, AEA and PEA.

### The female PD group is characterized by a reduction in inflammatory markers

**Figure 2:**
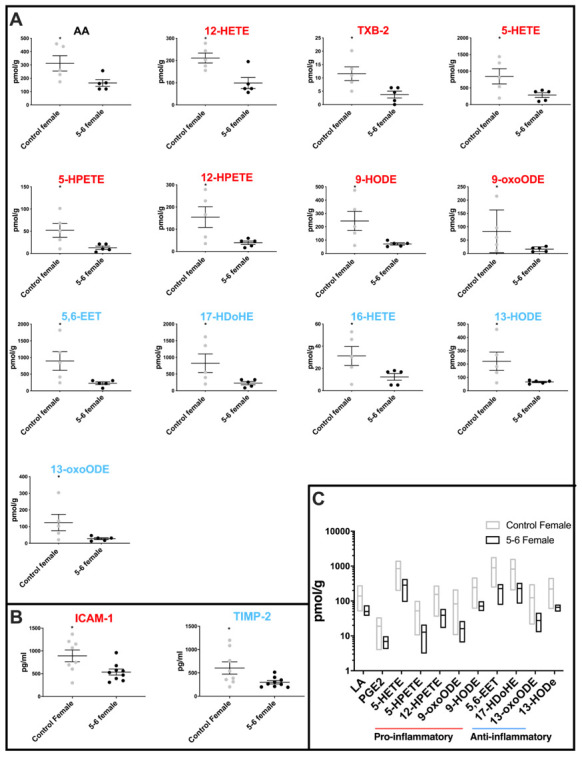
**Molecular profiling of inflammation in female PD cerebellar mitochondria.** (**A**) Oxylipin levels are lower in cerebellar mitochondria of PD Braak 5-6 females. Twelve oxylipin species and arachidonic acid are significantly reduced in the cerebellar mitochondria of females with PD Braak 5-6 when compared with controls. All samples were age matched. Braak 5-6 female n=5; control female n=5. (**B**) Two inflammatory cytokines are significantly lower in cerebellar mitochondria of PD Braak 5-6 females. ICAM-1 and TIMP-2 were significantly reduced in cerebellar mitochondria of PD Braak 5-6 females compared with female controls. Braak 5-6 female n=9; control female n=8. Plots display mean levels ± SEM. Red/blue titles represent pro- /anti-inflammatory, respectively. Refer to Supplementary Tables 3 and 5 for *p* values of oxylipins and inflammatory cytokines, respectively (Mann-Whitney U-test). (**C**) There is more variation in oxylipin content from cerebellar mitochondria of the female control group than the PD Braak 5-6 group. The female control group had significantly greater variance in eleven oxylipin quantities than PD Braak 5-6 females. PD Braak 5-6 female n=5; control female n=5. Box plots display interleaved high and low. The horizontal line represents the mean. Refer to Supplementary Table 4 for *f* values.

Comparison of cerebellar mitochondrial oxylipin levels in female PD Braak 5-6 fractions with controls shows a significant reduction in the levels of thirteen oxylipins (Figure 2A). AA and pro-inflammatory molecules12-HETE, TXB2, 5-HETE, 5-HPETE, 12-HPETE, 9-HODE and 9-oxoHODE are all significantly reduced in the PD females. Also, five oxylipins with anti-inflammatory roles were significantly reduced in the PD female samples these were 5,6-EET, 16-HETE, 17-HDoHE, 13-HODE and 13-oxoODE (see Supplementary Table 3 for *p values*).

Two cytokines, one pro-inflammatory ICAM-1 (*p=0.0274*) and the other anti-inflammatory TIMP-2 (*p=0.036*) were found to be significantly reduced in PD females when compared with controls (Figure 2B and Supplementary Table 5).

### Variation in levels of oxylipins in the female control group is attenuated in PD cerebellar mitochondria

Comparison of oxylipin levels in control and PD Braak 5-6 groups showed that the variation of oxylipin levels that characterized the female control cerebellar mitochondria is much reduced in the PD Braak 5-6 group. Eleven oxylipins showed a significant reduction in variation (*f*-test) in the PD group, these were LA and PGE2; pro-inflammatory molecules 5-HETE, 5-HPETE, 12-HPETE, 9-oxoODE, 9-HODE, 5,6-EET and17-HDoHE; anti-inflammatory oxylipins 13-oxoODE and 13-HODE (Figure 2C also Supplementary Figure 1 and Table 4).

### Free fatty acids and PTGS2 levels are significantly altered in male PD Braak 5-6 cerebellar mitochondrial fractions

**Figure 3:**
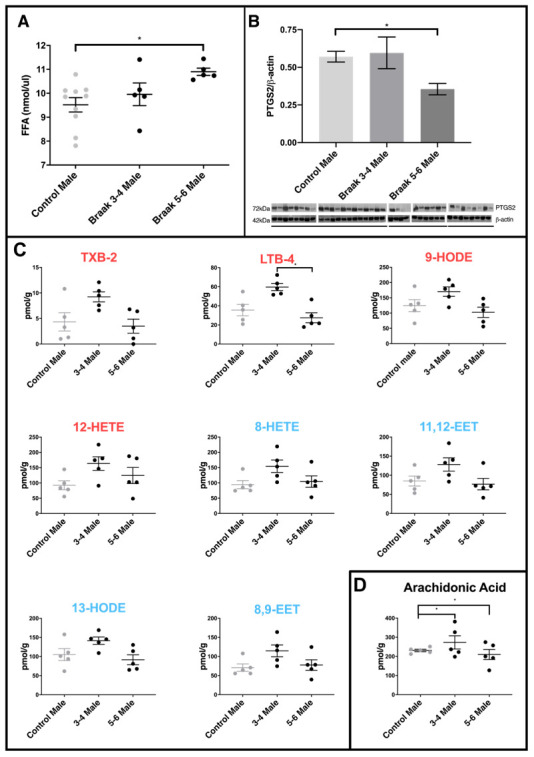
**Comparison of PD cerebellar mitochondria with different Braak classification.** (**A**) Free fatty acid concentration is increased in cerebellar mitochondria of PD Braak stage 5-6 males. A significant increase in FFA levels was shown in cerebellar mitochondria of Braak stage 5-6 males compared to age-matched controls (*p*=0.0164). No significant differences were apparent between PD Braak stage 3-4 and control. PD Braak 3-4 males (n=5), PD Braak 5-6 males (n=5), age-matched control males (n=10). Plots show mean values (nmol/μl) ± SEM (Kruskal-Wallis test with multiple comparisons). (**B**) Cerebellar levels of PTGS2 in PD Braak 5-6 mitochondrial fractions are lower compared with PD Braak 3-4 and controls. PTGS2 measured in enriched mitochondrial fractions were present in significantly lower quantities in the cerebellum of PD Braak 5-6 males compared to male controls (*p*=0.0307). No significant difference in PTGS2 levels were seen between PD Braak 3-4 and age-matched control. Mitochondrial PTGS2 levels, normalized to β-actin, were determined by Western blotting using mitochondrial and cytosolic fractions extracted from cerebellar tissue from age-matched male control (n=10) and PD Braak 3-4 (n=5) and 5-6 (n=4). Columns show the mean ±SEM (Kruskal-Wallis test with multiple comparisons). (**C**) Oxylipin concentrations in cerebellar mitochondria. LTB-4 is significantly decreased in PD Braak 5-6 males (n=5) compared to PD Braak 3-4 males (n=5); *p*=0.0139 (Kruskal-Wallis test with multiple comparisons). No significant differences were apparent between PD male groups and age-matched male controls (n=5). Red /blue titles represent pro- /anti-inflammatory, respectively. No significant changes were found between control males and PD Braak 5-6 males. Plots display mean concentration (pmol/g) ±SEM. Refer to Supplementary Table 5 for all *p* values (Kruskal-Wallis test with multiple comparisons). (**D**) Arachidonic acid levels in PD. Cerebellar mitochondrial arachidonic acid levels are similar in control males but significant variation is seen in PD Braak 3-4 and 5-6 males. Refer to Supplementary Table 4 for *f* values. All samples were age matched. PD Braak 3-4 male n=5; PD Braak 5-6 male n=5; control male n=5.

We measured total free fatty acid levels in the cerebellar mitochondria from control, PD Braak 3-4 (more moderate pathology) and PD Braak 5-6 brains. Though the numbers of samples were limited there was an upward trend in the PD Braak 3-4 samples, becoming a significant increase in free fatty acid content *(p=0.0164)* in the more severely affected PD Braak 5-6 derived samples (Figure 3A). The enzyme prostaglandin synthase 2 (PTGS2, also known as cyclooxygenase 2 or COX2) has a role in regulating inflammatory processes and is found to be increased in dopaminergic neurons of the *substantia nigra* in PD [35]. PTGS2 had not been previously measured in the cerebellar mitochondria of PD brains. In cerebellum samples we saw no change in PTGS2 levels in PD Braak 3-4 samples, however a significant drop in the levels of this enzyme in PD Braak 5-6 samples (*p*=0.0307) was measured (Figure 3B).

### A pattern of higher levels of oxylipins is seen in PDBraak 3-4 males

Many of the oxylipin species measured had a pattern of increased levels in Braak 3-4 male cerebellar mitochondria when compared with controls and Braak 5-6 samples (figure 3C). A selection of the species that exhibited this pattern are shown, including LTB4 where significance is reached between samples from PD Braak 3-4 and PD Braak 5-6 (*p=0.0139*).

### Arachidonic acid levels vary in PD cerebellar mitochondria

AA is metabolized through well characterized pathways to generate a range of oxylipin species [18]. In control males we see quite similar and consistent levels of AA across the samples that were analysed (Figure 3C). However, there is significantly more variation in AA levels in both the PD Braak 3-4 samples (*p=0.0072*) and the PD Braak 5-6 samples (*p=0.0209*) when compared with controls (for *f* -test values see Supplementary Table 4).

### Mean levels and variation of levels of cytokines are increased in male PD Braak 5-6 cerebellar mitochondria

**Figure 4:**
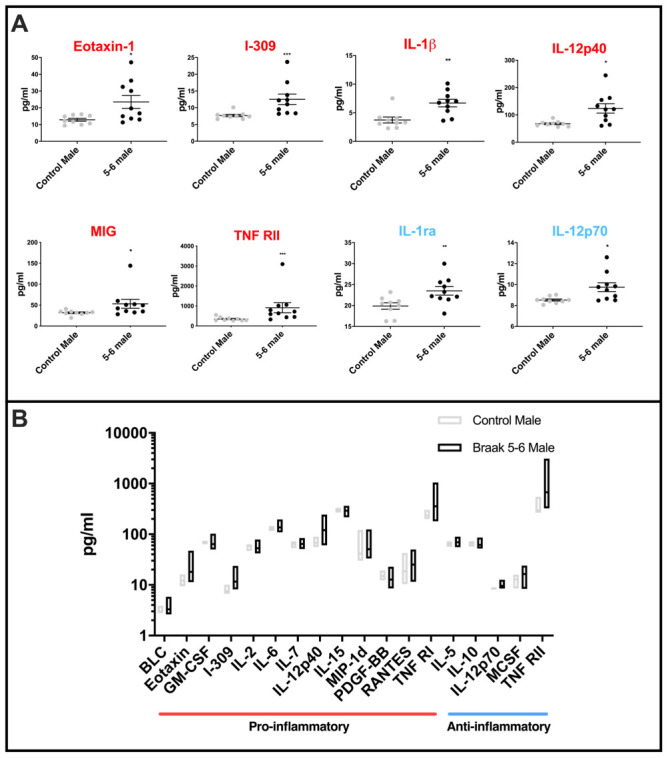
Cytokine levels in PD Braak 5-6 male cerebellar mitochondria (**A**) Mean levels of cytokines are higher in cerebellar mitochondria of PD Braak 5-6 males. Eight inflammatory cytokines were increased in cerebellar mitochondria of PD Braak 5-6 males compared with control males. PD Braak 5-6 male n=10; control male n=9. Red or blue titles represent pro- /anti-inflammatory, respectively. Plots display mean concentration (pg/ml) ± SEM (Mann-Whitney test), see Supplementary Table 5 for *p* values (Mann-Whitney U test). (**B**) PD Braak 5-6 males show different variance in cerebellar mitochondrial cytokines levels than was seen in the control male group. All seventeen measured inflammatory cytokines with significant differences in variance are more dispersed from the mean in PD Braak 5-6 males than control males. PD Braak 5-6 male n=10; PD Braak 5-6 female n=9; control male n=9; control female n=8. Box plots display interleaved high and low. The horizontal line represents the mean. Data shown are oxylipins (A+B) with significant variances (*f*-test). Refer to Supplementary Table 6 for *f* values.

Pro-inflammatory cytokines Eotaxin-1, I-309, IL-1β, IL-12p40, MIG, TNF RII, and anti-inflammatory IL-1ra and IL-12p70 were each found at significantly higher mean levels in the male PD Braak cerebellar mitochondria samples than in matched controls (Figure 4A: for *p values* see Supplementary Table 5). The variation across the PD Braak 5-6 group was also much greater than across the control group (Figure 4B and for *f-test* values see Supplementary Table 6). Pro-inflammatory cytokines BLC, Eotaxin, GM-CSF, I-309, IL-2, IL-6, IL-7, IL-12p40, IL-15, MIP-1d, PDGFB, RANTES, TNF RI, and anti-inflammatory cytokines IL-5, IL-10, IL-12p70, MCSF and TNF RII each had significantly greater variance in levels across the PD Braak 5-6 group compared with controls.

## DISCUSSION

Though much has been determined about PD associated changes in the cerebellum using imaging techniques, specific molecular and pathological evidence is scant [1, 36, 37]. Epigenetic changes in PD cerebellum have been investigated and give good reason to anticipate that regulatory pathways are changed during the disease course [6, 38]. Mitochondrial dysfunction is a key feature of PD. Our molecular analyses of mitochondrial fractions now show quite clearly that there are disease associated changes of inflammation pathways in PD cerebellum.

Inflammatory processes in the context of PD have suddenly been placed in the spotlight with the discovery that mitophagy proteins PINK1 and Parkin (both are implicated in familial PD) are important for regulating innate immunity and inflammation as shown in rodent models [11, 39]. We are the first to quantify molecular changes consistent with dysregulation of inflammatory processes in mitochondrial fractions of post-mortem human cerebellum. The role of neuroinflammation in PD is going to take some unravelling, but our data establish the cerebellum as a region that is affected in the disease process. Neuroinflammation has been shown to commence early in PD, peaking in moderately affected individuals, this fits with our data on PD Braak 3-4 subjects [40]. CSF and plasma DHA derived resolvin D1 (RvD1) are reduced in patients with early-onset PD [41], suggesting that inflammation resolution processes could be dysfunctional. We find the picture is complex, as should be expected, with both anti- and pro-inflammatory processes activated simultaneously to tune a potentially compensatory response to disease.

Sex differences in PD are recognized but have not been investigated thoroughly, particularly from a biochemical aspect [42–44]. Differences in development of the immune system and lifelong neuro-epigenetic differences in brain inflammatory profiles have been explored in rodents but the extent to which these findings translate to the human brain is not clear [45]. Specifically interesting in PD is the role of the testis-determining factor gene SRY [46]. In mouse, the SRY gene promotes catecholamine production by dopaminergic neurons of the substantia nigra [47], and rat models with repressed SRY expression show protection from experimentally induced PD [48]. A recent analysis shows an elevated prevalence of PD in females with the LRRK2 G2019S mutation [49]. Sexual dimorphism has been observed in the inflammatory response to traumatic brain injury in rodents [50, 51], and there is some limited information on sex differences in eicosanoid biology [52]. It is suggested that females can mount a more acute and efficient immune response, and subsequently resolve it more effectively than males [53–55]. The heterogeneity we see in the levels of inflammatory molecules in female controls could be responsible for the delay in onset and other phenotypic sex differences in PD. We found RANTES, a chemokine that induces infiltration into T-cells, to be significantly increased in female control cerebellar mitochondria compared with males. Decreasing RANTES in the *substantia nigra* has been shown to prevent loss of dopaminergic neurons in mouse models of PD [56]. In human PD, post-mortem *substantia nigra* contains more RANTES confirming association with disease but not necessarily with cell death in this region. From our study it is difficult to tell whether control females with low levels of the markers that are significantly more varied in the female control group, are at a higher risk for PD in the future. We show that sex differences are important to define in all studies of PD. Sex specific datasets can give clues about disease mechanism and pave the way to personalized medical approaches. Measurement of many markers of inflammatory processes in our study show that the control female groups have a greater range of these molecules with significantly higher mean levels. The greater range seen in females is interesting and needs to be explored further. It may be that younger females have generally high levels and the lower values seen in these age groups reflect a reduction associated with increased age. All this leads to the suggestion that neuroinflammatory responses measured as increases in oxylipins and cytokines, may be a protective state or acute response which changes or becomes exhausted in chronic disease states [57, 58]. This is potentially supported by the data shown for trends in the male groups where we were able to measure levels of oxylipins in control, PD Braak3-4 and PD Braak 5-6 cerebellar mitochondria. Though significance was only reached in the case of LTB-4 there was a trend towards an increase in levels of inflammatory molecules at the earlier disease stage (PD Braak 3-4), which was abated in the more severely affected PD Braak 5-6 group. The reduced range, or flat level of the molecules being measured in PD Braak 5-6 males is striking. This is a stage of disease that is clearly advanced with massive cell losses in the substantia nigra, it may be that the inflammatory response has been exhausted in the earlier stages of the disease. LTB-4 has been implicated previously in PD studies [59], but due to the poor correlation of rodent model PD to human disease it is difficult to draw any real conclusions about its precise role in the process.

Patterns of cytokine levels add further to the complexity of the picture of neuroinflammation in PD. Mean increases of both pro- and anti-inflammatory cytokines in cerebellar mitochondria could be used to define the male PD Braak 5-6 group. The most significant of these increases were in the pro-inflammatory molecules I-309 and TNF RII, followed by IL-1β and anti-inflammatory IL-1ra. TNF-RII antagonists have been proposed to have a therapeutic effect upon PD disease pathways, suggesting that this increase may be initiated in the cerebellum to compensate disease process in the *substantia nigra* [60]. Similar proposals have been made with respect to the cytokine I-309 [61]. There are a few studies on IL-1β and IL-1ra in the context of PD, supporting the idea of inflammatory responses in brain regions other than the *substantia nigra*.

We have compiled a molecular profile of inflammation in PD cerebellar mitochondria. This study emphasizes that that there are interesting molecular changes in the cerebellum that correlate with PD. Our findings highlight the need to establish sex specific differences in disease at the molecular level when these are recognized in the clinical phenotype.

## Materials and Methods

### Human post-mortem cerebellar samples

This study was performed with ethical approval from Parkinson’s UK Brain Bank and University of Nottingham SVMS Ethics committee (Reference# 2035 170519). Please see Supplementary Table 1 for information on patients and controls used in this study. Cerebellar mitochondria were isolated from 24 control, and 34 PD post-mortem brains. PD cases were further classified into Braak stages 3-4 and 5-6 respectively. Braak staging was assigned at the Parkinson’s UK Brain Bank according to published classification [62]. Oxylipin and cytokine profiling experiments used different post-mortem brain sample sets precluding sample group specific conclusions.

### Mitochondrial isolation

Enriched mitochondrial fractions were separated by differential centrifugation according to our previously published protocols [63]. Briefly, previously flash frozen cerebellar samples were placed in GentleMACS C tubes with mitochondria extraction buffer (50 mM Tris-HCl ph7.4, 100 mM KCl, 1.5 mM MgCl_2_, 1 mM EGTA, 50 mM HEPES and 100 mM sucrose; all sourced from Sigma-Aldrich, UK) and homogenized using a GentleMACS Dissociator (Miltenyi Biotec). The resulting homogenates were spun at 4°C in an Eppendorf Model 5417R Microcentrifuge (Fisher Scientific); first at 850 x g for 10 minutes, then the supernatant obtained was centrifuged separately at 1000 x g for 10 minutes to yield a nuclear pellet and a final spin at 10000 x g for 30 minutes to produce the mitochondrial pellet; the remaining supernatant contained the cytosolic fraction. Fractions were stored at -80°C.

### Protein assay

The protein concentrations of the samples were determined using the Bradford Assay - Sigma [64]. Bovine serum albumin (BSA- Fisher Scientific) standards of known protein concentration varying from 0 to 2mg/ml were added to Bradford reagent in disposable cuvettes (Fisher Scientific) and the absorbance was measured spectrophotometrically at 595 nm in a Thermo Scientific Spectrometer Helios Episilon (Fisher Scientific). The absorbance of the known protein standards was plotted using linear regression. The mitochondrial fractions were diluted 1 μl in 100 μl Tris Buffer and added to Bradford reagent before the absorbance was measured. The absorbance was plotted against the BSA standard curve to determine the estimated protein concentration (mg/ml).

### Western blot

Western blotting for quality control of fractions was carried out as described previously [65]. Antibody dilutions: PTGS2 ab62331 (Abcam) 1:2000 dilution in 5% (w/v) BSA in 1xTBS-T; β-actin ab8227 (Abcam) in 5% (w/v) BSA in 1xTBS-T; Goat anti-Rabbit IgG (HRP) A16096 (Invitrogen) 1:1000 in 5% (w/v) BSA in 1xTBS-T. Mitochondrial fractions were normalized to β-actin level. Blots were visualized using ChemiDoc MP Imaging System (Bio-Rad) and ECL Plus reagent (Pierce). Densitometry was carried out using ImageJ. Statistical analyses were carried out in GraphPad Prism.

### Free fatty acid quantitation

Total fatty acid concentration was measured using a Free Fatty Acid Quantification Kit (AB65341, Abcam), according to manufacturer’s instructions. Fatty acid concentrations were quantified by fluorometric analysis (Varioskan LUX Multimode Microplate Reader).

### Oxylipin quantitation

LC-ESI^—^MS/MS was used for analysis of oxylipins (AA, LA, 12-HETE, TXB-2, PGE2, 5-HETE, 5-HPETE, 12-HPETE, 9-HETE, 20-HETE, LTB-4, 9-oxoODE, 9-HODE, 5,6-EET, 5,6-DHET, 8,9-DHET, 11,12-DHET, 14,15-DHET, 8-HETE, 11-HETE, 15-HETE, 16-HETE, 8,9-EET, 11,12-EET, 14,15-EET, 17-HDoHE, 13-oxoODE, 13-HODE, 8,15-DiHETE) in mitochondrial fractions of human cerebellar tissue. A gradient LC method based on that described by Wong *et al* (14) using a modular Shimadzu Vp series HPLC was used to introduce the samples. Injection volume was 20μl. The MS system used was a triple quadrupole ion-trap 4000 QTRAP (Sciex, UK), equipped with Turbo Spray ionization interface. Standards were purchased from Cambridge Biosciences, Cambridge, UK. One batch of QC human plasma standard samples (from in-house stock) was used to confirm the intra-day accuracy of the method.

### Endocannabinoids quantitation

LC-ESI^—^MS/MS was used for analysis of endocannabinoids: arachidonyl ethanolamide (anandamide, AEA), 2-arachidonyl glycerol (2-AG), palmitoyl ethanolamide (PEA), oleoyl ethanolamide (OEA) in mitochondrial fractions of human cerebellar tissue. A uHPLC system, a modular Exion series LC (Sciex, Warrington, UK) was used to create a 10 min gradient and introduce the 5μl sample. The column (uPLC BEH C18 1.7μm (2.1x150mm, Waters, Elstree, UK) was held at 60°C [66]. The MS system used was a Qtrap 6500+ (Sciex, Warrington, UK) equipped with an electrospray ionization (ESI) interface. Standards were purchased from Cambridge Biosciences, Cambridge, UK. One batch of QC human plasma standard samples (from in-house stock) was used to confirm the intra-day accuracy of the method.

### CHuman inflammation antibody array

Abcam human inflammation antibody array (ab197451) kit was used for the detection of 40 cytokines: BLC, eoxtaxin-1, eotaxin-2, G-CSF, GM-CSF, I-309, ICAM-1, IFNγ, IL-1α, IL-1β, IL-1ra, IL-2, IL-4, IL-5, IL-6, IL-6sR, IL-7, IL-8, IL-10, IL-11, IL-12p40, IL-12p70, IL-13, IL-15, IL-16, IL-17. MCP-1, MCSF, MIG, MIP-1α, MIP-1β, MIP-1δ, PDGF-BB, RANTES, TIMP-1, TIMP-2, TNFα, TNFβ, sTNF RI, sTNF RII (guide to abbreviations in Supplementary Table 2). Glass slides contained 16 identical antibody arrays, with a 16 well gasket for addition of separate samples to each array. Antibodies were spotted in quadruplicate in each array. Protocol was followed as per manufacturer’s instructions. Briefly, gasket wells were blocked for 30 minutes in Sample Diluent, followed by addition of a standard cytokine cocktail or 100ug of sample, diluted in PBS. The cocktail of standard cytokines was diluted three-fold across eight dilutions and 100ul was added to each well for 60 minutes. Slides were washed in supplied wash buffer 7 times for 5 minutes each, with gentle agitation at room temperature. The detection antibody was reconstituted in 1.4ml sample diluent and 80ul added to each well and incubated overnight at 4°C, with gentle agitation. Subsequently, another 7 washes were carried out, as described above. 80ul of Cy3 equivalent dye-conjugated streptavidin was added to each well. At this step, the slide was covered in aluminium foil to avoid exposure to light and incubated at room temperature for 1 hour. A further 5 wash steps for 5 minutes each followed. A final 2 wash steps were carried out for 15 minutes each. Finally, slides were completely dried with 3 times 3-minute centrifugation steps at 1,000rpm. For signal visualisation, slides were scanned at 532nm and data analysed using GenePix Pro Software (Axon GenePix). The median local background was subtracted from the median fluorescence of each spot and the corrected fluorescence was used to calculate the average fluorescence signal as well as the standard deviation.

### Statistical analysis

Data from the various experiments were presented as mean average alongside calculated standard deviation and standard error mean. Mann-Whitney U and Kruskal-Wallis tests were performed on the datasets using GraphPad Prism version 6 for Windows (GraphPad Software, San Diego California USA).

## Supplementary Material

Supplementary Figures

Supplementary Tables
