# Supplementary material for: Sex specific inflammatory profiles of cerebellar mitochondria are attenuated in Parkinson’s disease
Source: Aging (Albany NY). 2020 Aug 27;12(17):17713–37. doi: 10.18632/aging.103937 (PMC7521528; doi:10.18632/aging.103937)
Supplement: Supplementary Figures [file aging-12-103937-s001..pdf]

SUPPLEMENTARY FIGURES

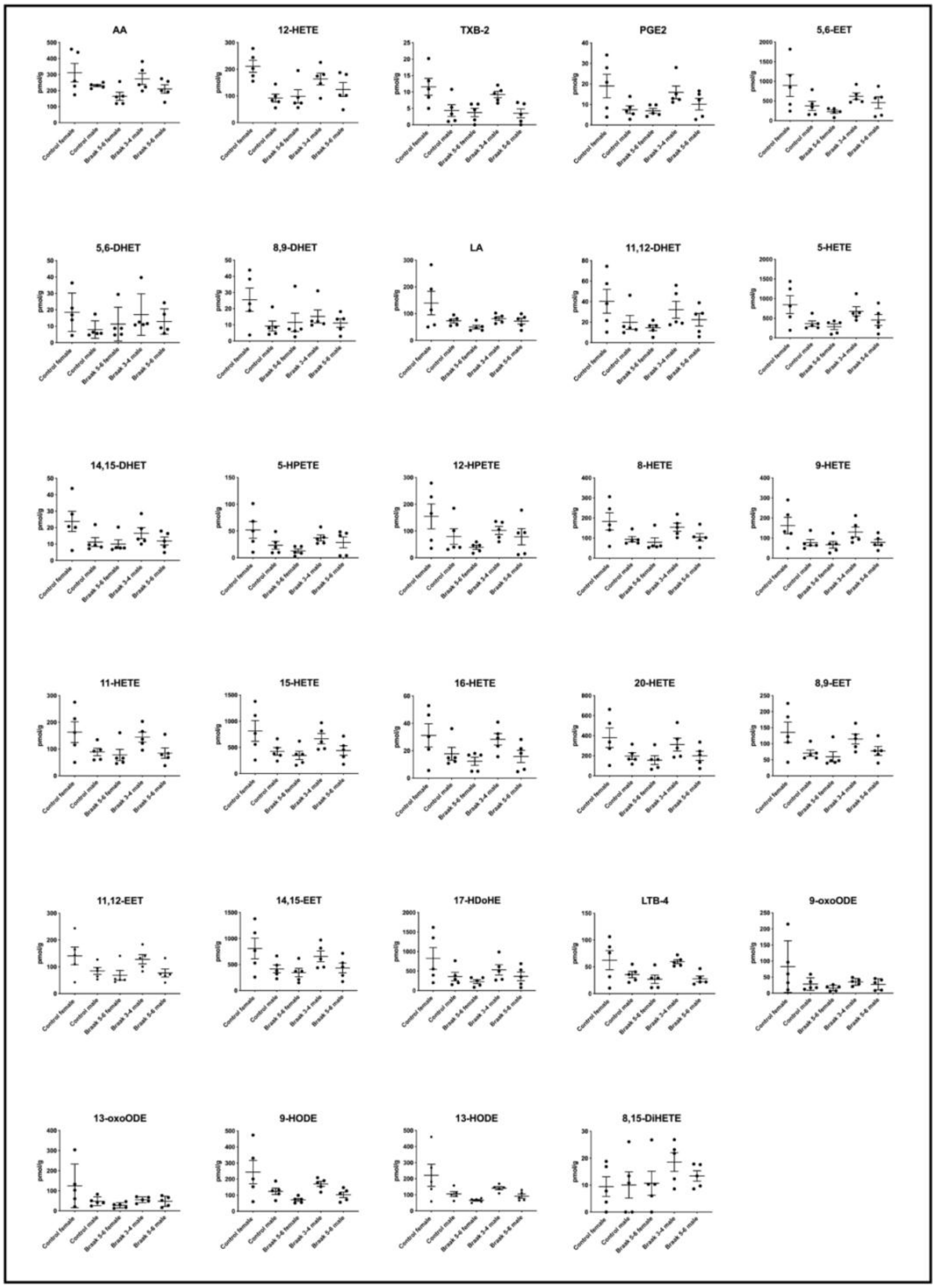

Supplementary Figure 1. A large variation of oxylipin concentration is seen in the control female group. Comparison of measured oxylipins in cerebellar mitochondria from PD males (Braak 3-4 and 5-6) and PD females (Braak 5-6) and age-matched controls. PD Braak 3-4 male n=5; PD Braak 5-6 male n=5; PD Braak 5-6 female n=5; control male n=5; control female n=5. Plots display mean quantities (pmol/g) ± SEM. For *p* and *f* values refer to Supplementary Tables 3 and 4.

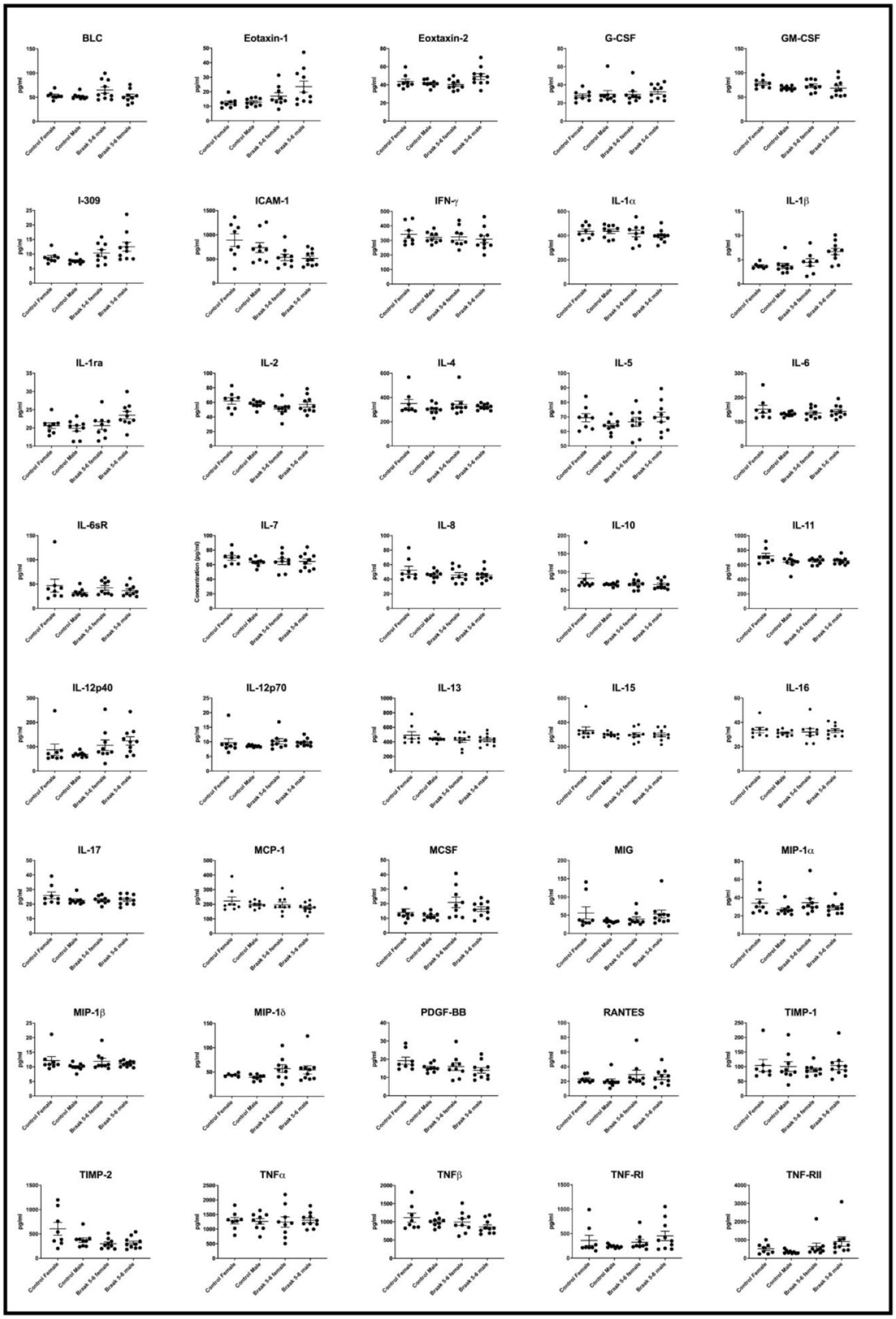

Supplementary Figure 2. Graphical comparisons of all measured inflammatory cytokines from cerebellar mitochondria of males and females with Braak stage 5-6 Parkinson's disease and age-matched controls. PD Braak 5-6 male n=10; PD Braak 5-6 female n=9; control male n=9; control female n=8. Plots display mean concentration (pg/ml) ± SEM. For all *p* or *f* values refer to Supplementary Tables 5 or 6, respectively.
