# Supplementary material for: Sex specific inflammatory profiles of cerebellar mitochondria are attenuated in Parkinson’s disease
Source: Aging (Albany NY). 2020 Aug 27;12(17):17713–37. doi: 10.18632/aging.103937 (PMC7521528; doi:10.18632/aging.103937)
Supplement: Supplementary Tables [file aging-12-103937-s002..pdf]

SUPPLEMENTARY TABLES

Supplementary Table 1. Human post-mortem samples information.

ID	Diagnosis	Age (yr)	Sex	Disease duration (yr)	α-syn	Cause of death	Post mortem delay (hr)
75/02	Control	61	F	N/A	N/A	Not reported	N/A
CO48	Control	68	M	N/A	N/A	Metastatic colon cancer	10
CO22	Control	69	F	N/A	N/A	Lung cancer	33
PDC008	Control	71	F	N/A	N/A	Myocardial infarction	17
PDC030	Control	77	M	N/A	N/A	Conductive cardiac failure	17
CO45	Control	77	M	N/A	N/A	Multiple (old age)	22
CO26	Control	78	F	N/A	N/A	Myeloid leukaemia	33
61/07	Control	81	M	N/A	N/A	Not reported	N/A
PDC029	Control	82	M	N/A	N/A	Metastatic liver/lung cancer	48
PDC005	Control	58	M	N/A	N/A	Not reported	9
PDC022	Control	65	M	N/A	N/A	Lung carcinoma	12
PDC034	Control	90	M	N/A	N/A	Respiratory failure	12
PDC016	Control	93	F	N/A	N/A	Bronchial pneumonia/old	22
PDC026	Control	80	F	N/A	N/A	Breast carcinoma	23
PDC023	Control	78	F	N/A	N/A	Not reported	23
PDC008	Control	71	F	N/A	N/A	Myocardial infarction	17
PDC028	Control	84	F	N/A	N/A	Pancreatic cancer	11
PDC027	Control	79	M	N/A	N/A	Cardiac arrest & pneumonia	21
PDC092	Control	79	M	N/A	N/A	Brainstem stroke, bronchopneumonia	25
PDC014	Control	64	M	N/A	N/A	Cardiac failure	18
PDC078	Control	91	M	N/A	N/A	Not reported	18
PDC105	Control	95	M	N/A	N/A	Pneumonia, cellulitis of leg	11
PDC053	Control	89	F	N/A	N/A	Not reported	22
PDC040	Control	61	F	N/A	N/A	Ovarian cancer	15
PD081	Braak 3-4	73	M	9	6	Not reported	19
PD036	Braak 3-4	76	M	10	3	Not reported	10
PD041	Braak 3-4	77	M	10	6	Not reported	6
PD007	Braak 3-4	78	M	10	3	Pneumonia	22
PD067	Braak 3-4	83	M	9	6	Not reported	10
PD051	Braak 3-4	80	M	5	5	Not reported	7
PD067	Braak 3-4	83	M	9	N/A	Not reported	10
PD081	Braak 3-4	73	M	9	N/A	Not reported	19
PD084	Braak 3-4	78	M	9	5	Ischaemic bowel and atrial fibrillation	3
PD074	Braak 3-4	85	M	5	5	bronchopneumonia	7
002/08	Braak 5-6	69	M	1	Not available	Not reported	N/A
PD131	Braak 5-6	76	F	11	6	Not reported	22
044/07	Braak 5-6	78	M	6	Not available	Not reported	N/A
PD014	Braak 5-6	79	M	12	3	Parkinson’s	21
PD063	Braak 5-6	80	F	13	4	Old age and PD	10
PD028	Braak 5-6	82	M	18	6	Not reported	14
PD050	Braak 5-6	82	F	14	6	Chest infection & CVA	18
PD099	Braak 5-6	82	M	11	6	Pneumonia	10
PD016	Braak 5-6	85	F	18	6	Bronchopneumonia/PD	14
PD045	Braak 5-6	80	M	19	6	Not reported	16
PD028	Braak 5-6	82	M	18	6	Not reported	14
PD079	Braak 5-6	78	F	19	6	Chest infection & CVA	22
PD016	Braak 5-6	85	F	18	6	Bronchopneumonia/PD	14
PD125	Braak 5-6	74	M	25	6	Bronchopneumonia/PD	20
PD117	Braak 5-6	77	F	31	5	Not reported	6
PD021	Braak 5-6	76	M	27	6	Not reported	17
PD020	Braak 5-6	75	M	34	6	Not reported	2

PD117	Braak 5-6	77	F	31	5	Not reported	6
PD017	Braak 5-6	75	M	18	6	Not reported	22
PD180	Braak 5-6	85	F	15	6	Chest infection & PD	15
PD093	Braak 5-6	81	F	14	6	Not reported	22
PD203	Braak 5-6	84	F	18	6	PD	19
PD413	Braak 5-6	72	F	21	6	Pneumonia	14

Supplementary Table 2. Abbreviations and corresponding full names of all oxylipins, inflammatory cytokines and endocannabinoids used in this work.

Abbreviation	
Oxlipins	
AA	Arachidonic acid
LA	Linoleic acid
12-HETE	12-hydroxyeicosatetraenoic acid
TXB-2	Thromboxane B2
PGE2	Prostaglandin E_2
5-HETE	5-Hydroxyeicosatetraenoic acid
5-HPETE	Arachidonic acid 5-hydroperoxide
12-HPETE	Arachidonic acid 12-hydroperoxide
9-HETE	9-Hydroxyeicosatetraenoic acid
20-HETE	20-Hydroxyeicosatetraenoic acid
LTB-4	Leukotriene B4
9-oxoODE	9-oxo-10E,12Z-octadecadienoic acid
9-HODE	9S-Hydroxy-10E,12Z-octadecadienoic acid
5,6-EET	5,6-epoxyeicosatrienoic acid
5,6-DHET	5,6-dihydroxyeicosatrienoic acid
8,9-DHET	8,9-dihydroxyeicosatrienoic acid
11,12-DHET	11,12-dihydroxyeicosatrienoic acid
14,15-DHET	14,15-dihydroxyeicosatrienoic acid
8-HETE	8-Hydroxyeicosatetraenoic acid
11-HETE	11-Hydroxyeicosatetraenoic acid
15-HETE	15-Hydroxyeicosatetraenoic acid
16-HETE	16-Hydroxyeicosatetraenoic acid
8,9-EET	8,9-epoxyeicosatrienoic acid
11,12-EET	11,12-epoxyeicosatrienoic acid
14,15-EET	14,15-epoxyeicosatrienoic acid
17-HDoHE	17-Hydroxydocosahexaenoic acid
13-oxoODE	13-Oxo-9,11-octadecadienoic acid
13-HODE	13-hydroxy-9Z,11E-octadecadienoic acid
8,15-DiHETE	8,15-Leukotriene B4
Endocannabinoids	
2-AG	2-Arachidonoylglycerol
AEA	Anandamide
OEA	Oleylethanolamide
PEA	Palmitoylethanolamide
Inflammatory cytokines	
BLC	B Cell-Attracting Chemokine 1
Eotaxin	C-C Motif Chemokine Ligand 11 (CCL11)
Eotaxin-2	C-C Motif Chemokine Ligand 24 (CCL24)
G-CSF	Granulocyte Colony-Stimulating Factor
GM-CSF	Granulocyte-Macrophage Colony-Stimulating Factor

I-309	C-C Motif Chemokine Ligand 1 (CCL1)
ICAM-1	Intercellular Adhesion Molecule 1
IFNγ	Interferon Gamma
IL-1α	Interleukin 1 Alpha
IL-1β	Interleukin 1 Beta
IL-2	Interleukin 2
IL-6	Interleukin 6
IL-6sR	Interleukin 6 soluble receptor
IL-7	Interleukin 7
IL-8	Interleukin 8
IL-12p40	IL-12 Subunit P40
IL-12p70	IL-12 Subunit P70
IL-15	Interleukin 15
IL-16	Interleukin 16
IL-17	Interleukin 17
MCP-1	Monocyte Chemotactic And Activating Factor
MIG	Monokine Induced By Interferon-Gamma
MIP-1α	Macrophage Inflammatory Protein 1-Alpha
MIP-1β	Macrophage Inflammatory Protein 1-Beta
MIP-1δ	Macrophage Inflammatory Protein 1-Delta
PDGF-BB	Platelet Derived Growth Factor Subunit B
RANTES	Regulated Upon Activation, Normally T-Expressed, And Secreted
TNFα	Tumor Necrosis Factor-Alpha
TNFβ	Tumor Necrosis Factor-Beta
TNF RI	Tumor Necrosis Factor Receptor 1
TNF RII	Tumor Necrosis Factor Receptor 2
IL-1ra	Interleukin 1 Receptor Antagonist
IL-4	Interleukin 4
IL-5	Interleukin 5
IL-10	Interleukin 10
IL-11	Interleukin 11
IL-13	Interleukin 13
MCSF	Macrophage Colony-Stimulating Factor 1
TIMP-1	Tissue Inhibitor Of Metalloproteinases 1
TIMP-2	Tissue Inhibitor Of Metalloproteinases 2

Supplementary Table 3. Mean comparisons of all 29 measured oxylipins from cerebellar mitochondria.

Oxylipin	Control males vs control females	Braak 5-6 males vs Braak 5-6 females	Braak 5-6 males vs control males	Braak 3-4 males vs control males	Braak 3-4 males vs Braak 5-6 males	Braak 3-4 males vs control females	Braak 5-6 females vs control females
AA	0.4206	0.1429	>0.9999	>0.9999	0.688	0.6905	**0.0317***
LA	0.4206	0.1984	>0.9999	>0.9999	>0.9999	0.6905	0.0556
12-HETE	**0.0079****	0.5159	0.5373	0.1209	>0.9999	0.2222	**0.0317***
TXB-2	0.0556	0.8571	>0.9999	0.1687	0.0589	0.5476	**0.0317***
PGE2	0.2222	0.6032	>0.9999	0.198	0.7737	>0.9999	0.2778
5-HETE	0.2222	0.3968	>0.9999	0.1687	0.6093	0.6905	**0.0476***
5-HPETE	0.0952	0.381	>0.9999	>0.9999	0.8665	0.5476	**0.0317***
12-HPETE	0.3095	0.6508	0.6905	0.3095	0.5476	0.5476	**0.0476***
9-HETE	0.2222	0.8016	>0.9999	0.1431	0.3116	0.5476	0.0873
20-HETE	0.2222	0.5238	>0.9999	0.4127	0.6093	0.8413	0.0873
LTB-4	0.4206	0.7857	>0.9999	0.1204	**0.0139***	0.6905	0.2063
9-oxoODE	0.3095	0.1905	>0.9999	>0.9999	>0.9999	0.4206	**0.0476***
9-HODE	0.2222	0.1905	>0.9999	0.3339	0.0774	0.6905	**0.0476***
5,6-EET	0.1508	0.381	>0.9999	0.4719	>0.9999	0.8413	**0.0317***
5,6-DHET	0.3095	0.5238	0.6081	0.1311	>0.9999	0.5476	0.381
8,9-DHET	0.2222	0.5397	>0.9999	0.5373	>0.9999	0.3095	0.2063
11,12-DHET	0.2222	0.3968	>0.9999	0.3116	0.7737	0.5476	0.1349
14,15-DHET	0.3095	0.5159	>0.9999	0.2691	0.6093	0.4206	0.1984
8-HETE	0.2222	0.381	>0.9999	0.0589	0.2691	0.6905	0.0873
11-HETE	0.2222	0.5	>0.9999	0.1687	0.1017	0.8413	0.1349
15-HETE	0.1508	0.5238	>0.9999	0.3116	0.3594	0.6905	0.0873
16-HETE	0.3095	0.3968	>0.9999	0.2313	0.198	0.8413	**0.0476***
8,9-EET	0.2222	0.2778	>0.9999	0.071	0.3594	0.6905	0.0873
11,12-EET	0.2222	0.5159	>0.9999	0.198	0.0851	0.6905	0.1984
14,15-EET	0.1508	0.5238	>0.9999	0.4127	0.6093	0.6905	0.0873
17-HDoHE	0.2222	0.381	>0.9999	>0.9999	>0.9999	0.6905	**0.0476***
13-oxoODE	0.2222	0.2857	>0.9999	>0.9999	>0.9999	0.3095	**0.0476***
13-HODE	0.2222	0.1905	>0.9999	0.2691	0.1017	0.5476	**0.0476***
8,15-DiHETE	>0.9999	0.5238	>0.9999	0.5338	>0.9999	0.0952	0.8968

PD Braak 3-4 male n=5; PD Braak 5-6 male n=5; PD Braak 5-6 female n=5; control male n=5; control female n=5. Statistical analyses were carried out using GraphPad Prism (Kruskal-Wallis test with multiple comparisons or Mann-Whitney U-test where appropriate). Red and blue font oxylipins represent pro- and anti-inflammatory, respectively. Bright yellow shaded numbers refer to significance (*p<0.05; **p<0.01).

Supplementary Table 4. Group variance analyses of all 29 measured oxylipins from cerebellar mitochondria.

Oxylipin	Control males vs control females	Braak 5-6 males vs Braak 5-6 females	Braak 5-6 males vs control males	Braak 3-4 males vs control males	Braak 3-4 males vs Braak 5-6 males	Braak 3-4 males vs control females	Braak 5-6 females vs control females
**AA**	**0.001****	0.9319	**0.0209***	**0.0072****	0.5974	0.3514	0.1354
**LA**	**0.0048****	0.3855	0.4618	0.9381	0.4176	**0.0041****	**0.0036****
**12-HETE**	0.4458	0.9151	0.2903	0.453	0.7467	0.9901	0.8379
**TXB-2**	0.5039	0.903	0.6142	0.2706	0.5359	0.0895	0.2078
**PGE2**	0.0517	0.125	0.4369	0.349	0.8676	0.2616	**0.0104***
**5-HETE**	**0.0402***	0.2352	0.2075	0.3246	0.7657	0.2306	**0.0468***
**5-HPETE**	0.1729	0.0686	0.538	0.6102	0.2696	0.0715	**0.0149***
**12-HPETE**	0.3939	**0.0208***	0.9326	0.2237	0.1959	0.0503	**0.0043****
**9-HETE**	0.0878	0.8281	0.948	0.3884	0.3553	0.3558	0.1145
**20-HETE**	0.0641	0.9358	0.5881	0.2454	0.5188	0.4326	0.1447
**LTB-4**	0.0567	0.454	0.7851	0.3996	0.5632	**0.0108***	0.1338
**9-oxoODE**	**0.0173***	0.2025	0.8634	0.2913	0.3717	**0.0019****	**0.0008*****
**9-HODE**	**0.0272***	0.2072	0.7879	0.6756	0.8803	**0.0119***	**0.0011****
**5,6-EET**	0.1154	**0.0308***	0.6494	0.5482	0.2996	**0.0385***	**0.0027****
**5,6-DHET**	0.1545	0.5922	0.4865	0.1216	0.3628	0.8868	0.8066
**8,9-DHET**	0.1304	0.1762	0.7949	0.6324	0.4639	0.28	0.6597
**11,12-DHET**	0.3047	0.158	0.8692	0.7037	0.5873	0.5078	0.0169
**14,15-DHET**	0.137	0.9187	0.8623	0.669	0.5498	0.2709	0.1214
**8-HETE**	**0.0431***	0.8093	0.516	0.4253	0.8786	0.1791	0.2063
**11-HETE**	0.0707	0.8643	0.535	0.5844	0.9402	0.1819	0.2665
**15-HETE**	0.0759	0.8024	0.6538	0.5574	0.8881	0.2066	0.108
**16-HETE**	0.268	0.4558	0.9429	0.8615	0.9181	0.2051	0.0674
**8,9-EET**	**0.0408***	0.8163	0.5151	0.3884	0.8263	0.1907	0.1947
**11,12-EET**	0.0946	0.7192	0.7711	0.5743	0.7844	0.2406	0.274
**14,15-EET**	0.0859	0.7794	0.675	0.5518	0.8582	0.2331	0.1098
**17-HDoHE**	0.0951	0.1531	0.9555	0.7221	0.764	0.174	**0.006****
**13-oxoODE**	**0.0085****	0.2321	0.7652	0.4311	0.2845	**0.0016****	**0.0012****
**13-HODE**	**0.0138***	0.1104	0.7363	0.3799	0.5811	**0.0022****	**0.0002*****
**8,15-DiHETE**	0.5978	0.1323	0.0999	0.5292	0.2795	0.9173	0.7143

PD Braak 3-4 male n=5; PD Braak 5-6 male n=5; PD Braak 5-6 female n=5; control male n=5; control female n=5. Statistical analyses were carried out using GraphPad Prism (*f*-test). Red and blue font oxylipins represent pro- and anti-inflammatory, respectively. Bright yellow shaded numbers refer to significance (*p<0.05; **p<0.01; ***p<0.001).

Supplementary Table 5. Statistical comparisons of all 40 measured inflammatory cytokines from cerebellar mitochondria.

Inflammatory Cytokine	Control males vs control females	Braak 5-6 males vs Braak 5-6 females	Braak 5-6 males vs control males	Braak 5-6 females vs control females
BLC	0.4807	0.1564	0.1823	0.5414
Eotaxin	0.6058	0.4363	**0.04***	0.0745
Eotaxin-2	0.8884	0.077	0.0503	0.3704
G-CSF	0.8884	0.3562	0.3154	>0.9999
GM-CSF	**0.0418***	0.4002	0.6607	>0.9999
I-309	0.0745	0.5457	**0.0005*****	0.4234
ICAM-1	0.4234	>0.9999	0.0789	**0.0274***
IFNγ	0.8148	0.447	0.3562	0.6058
IL-1α	0.9626	0.447	0.2428	0.743
IL-1β	0.536	0.0545	**0.0057****	0.3357
IL-2	0.3213	0.447	0.7197	0.1388
IL-6	0.4807	0.6607	0.549	0.5414
IL-6sR	0.743	0.3562	0.6038	0.5414
IL-7	0.3213	0.9048	0.9682	0.6058
IL-8	0.743	0.9048	0.549	0.3213
IL-12p40	0.8148	0.4894	**0.0188***	0.1672
IL-15	0.2766	0.9048	0.8421	0.3213
IL-16	0.5414	0.8421	0.4967	0.6058
IL-17	0.2359	0.8421	0.9048	0.6058
MCP-1	0.8148	0.4002	0.1333	0.4807
MIG	0.4807	0.2581	**0.0188***	0.8148
MIP-1α	0.1388	0.3154	0.3154	>0.9999
MIP-1β	0.0927	>0.9999	0.0653	0.8148
MIP-1δ	0.1812	0.4967	0.1728	0.2721
PDGF-BB	0.1139	0.6038	0.3154	0.2359
RANTES	**0.007****	0.9682	0.1011	0.6058
TNFα	0.9626	0.6607	0.9048	0.5414
TNFβ	0.9626	0.3154	0.0789	0.6058
TNF RI	0.9626	0.4967	0.1564	0.3704
TNF RII	0.3213	0.2224	**0.0012****	0.9626
IL-1ra	0.6058	0.0535	**0.0057****	0.8884
IL-4	0.5414	0.9682	0.1823	0.8148
IL-5	0.2359	0.4967	0.1823	0.673
IL-10	0.4234	0.7802	0.6607	0.6058
IL-11	0.1672	0.9048	0.7802	0.1996
IL-12p70	0.743	>0.9999	**0.0101***	0.3213
IL-13	0.673	0.9682	0.6607	0.8148
MCSF	0.743	0.4967	0.0653	0.1996
TIMP-1	0.9182	0.4967	0.6607	0.9182
TIMP-2	0.3704	0.7802	0.2428	**0.036***

PD Braak 5-6 male n=10; PD Braak 5-6 female n=9; control male n=9; control female n=8. Statistical analyses were carried out using GraphPad Prism (Mann-Whitney U-test). Red and blue font represent pro- and anti-inflammatory, respectively. Bright yellow shaded numbers refer to significance (*p<0.05; **p<0.01; ***p<0.001).

Supplementary Table 6. Statistical group variance analyses of all 40 measured inflammatory cytokines from cerebellar mitochondria.

Inflammatory Cytokine	Control males vs control females	Braak 5-6 males vs Braak 5-6 females	Braak 5-6 males vs control males	Braak 5-6 females vs control females
BLC	0.489	0.2784	**0.0024****	0.1595
Eotaxin	0.4921	0.4269	**0.0017****	0.0723
Eotaxin-2	0.0547	0.4614	0.0586	0.429
G-CSF	0.0785	0.5697	0.269	0.1925
GM-CSF	0.1593	0.2312	**0.0004*****	0.2614
I-309	0.114	0.7636	**0.0054****	0.1258
ICAM-1	0.6282	0.4518	0.0546	0.1108
IFNγ	0.0864	0.7262	0.057	0.8382
IL-1α	0.9418	0.1935	0.8813	0.321
IL-1β	**0.0221***	0.8982	0.4273	**0.0042****
IL-2	**0.0155***	0.7287	**0.0202***	0.5979
IL-6	**0.0001*****	0.7721	**0.0065****	0.0755
IL-6sR	**0.0002*****	0.5675	0.2664	**0.0145***
IL-7	0.1184	0.8129	**0.0419***	0.5199
IL-8	**0.0117***	0.6105	0.2609	0.2745
IL-12p40	**<0.0001******	0.129	**0.0013****	0.9745
IL-15	**0.0007*****	0.728	**0.0263***	0.2327
IL-16	**0.0166***	0.1326	0.0653	0.4724
IL-17	**0.0279***	0.3974	0.5947	**0.0138***
MCP-1	**0.0028****	0.1306	0.3733	0.3504
MIG	**<0.0001******	0.1617	0.0902	**0.0155***
MIP-1α	0.0575	**0.0419***	0.7264	0.7378
MIP-1β	**0.0044****	**0.0023****	0.5406	0.5712
MIP-1δ	0.2335	0.732	**0.0006*****	**0.0005*****
PDGF-BB	**0.0203***	0.4083	**0.0396***	0.6927
RANTES	0.3007	0.1473	**0.0046****	**0.0026****
TNFα	0.8404	0.0413*	0.7458	0.1598
TNFβ	**0.0155***	0.2411	0.3658	0.569
TNF RI	**<0.0001******	0.1133	**<0.0001******	0.1464
TNF RII	**0.0079****	0.0696	**0.0123***	**0.0479***
IL-1ra	0.9013	0.998	0.362	0.3329
IL-4	**0.0349***	**0.0005*****	0.0917	0.8174
IL-5	0.1335	0.7278	**0.0301***	0.755
IL-10	**<0.0001******	0.6593	**0.0095****	**0.0097****
IL-11	0.5622	0.7733	0.1148	**0.024***
IL-12p70	**<0.0001******	**0.0408***	**0.0004*****	0.337
IL-13	**0.0072****	0.5229	0.1929	0.3199
MCSF	**0.0052****	**0.0439***	**0.0367***	0.3129
TIMP-1	0.793	**0.0265***	0.7116	**0.0087****
TIMP-2	**0.0139***	0.7664	0.6342	**0.0022****

PD Braak 5-6 male n=10; PD Braak 5-6 female n=9; control male n=9; control female n=8. Statistical analyses were carried out using GraphPad Prism (*f*-test). Red and blue font represent pro- and anti-inflammatory cytokines, respectively. Bright yellow shaded numbers refer to significance (**p*<0.05; ***p*<0.01; ****p*<0.001; *****p*<0.0001).

Supplementary Table 7. Statistical comparisons of endocannabinoids were carried out using GraphPad Prism (Kruskal-Wallis and *f* tests).

Endocannabinoid	Control males vs control females	Braak 5-6 males vs Braak 5-6 females	Braak 5-6 females vs control females	Braak 5-6 males vs control males
p-values				
2-AG	>0.9999	>0.9999	>0.9999	>0.9999
AEA	>0.9999	>0.9999	>0.9999	>0.9999
OEA	>0.9999	>0.9999	0.9232	>0.9999
PEA	>0.9999	>0.9999	>0.9999	>0.9999
f-values				
2-AG	0.1801	**0.0441***	**0.0336***	0.2331
AEA	0.0829	0.9996	**0.0164***	0.5157
OEA	0.733	0.8458	0.6317	0.9572
PEA	0.7027	0.0814	**0.0397***	0.9634

Bright yellow shaded numbers refer to significance (*p<0.05). Braak 5-6 male n=8; Braak 5-6 female n=9; control male n=8; control female n=6. All samples were age matched.
